# Eighty routes to a ribonucleotide world; dispersion and stringency in the decisive selection

**DOI:** 10.1261/rna.066761.118

**Published:** 2018-08

**Authors:** Michael Yarus

**Affiliations:** Department of Molecular, Cellular and Developmental Biology, University of Colorado Boulder, Boulder, Colorado 80309-0347, USA

**Keywords:** RNA, biogenesis, genetics, expression, replication

## Abstract

We examine the initial emergence of genetics; that is, of an inherited chemical capability. The crucial actors are ribonucleotides, occasionally meeting in a prebiotic landscape. Previous work identified six influential variables during such random ribonucleotide pooling. Geochemical pools can be in periodic danger (e.g., from tides) or constant danger (e.g., from unfavorable weather). Such pools receive Gaussian nucleotide amounts sporadically, at random times, or get varying substrates simultaneously. Pools use cross-templated RNA synthesis (5′–5′ product from 5′–3′ template) or para-templated (5′–5′ product from 5′–5′ template) synthesis. Pools can undergo mild or strong selection, and be recently initiated (early) or late in age. Considering >80 combinations of these variables, selection calculations identify a superior route. Most likely, an early, sporadically fed, cross-templating pool in constant danger, receiving ≥1 mM nucleotides while under strong selection for a coenzyme-like product, will host selection of the first encoded biochemical functions. Predominantly templated products emerge from a critical event, the starting bloc selection, which exploits inevitable differences among early pools. Favorable selection has a simple rationale; it is increased by product dispersion (SD/mean), by selection intensity (mild or strong), or by combining these factors as stringency, reciprocal fraction of pools selected (1/*sf*_sel_). To summarize: chance utility, acting via a preference for disperse, templated coenzyme-like dinucleotides, uses stringent starting bloc selection to quickly establish majority encoded/genetic expression. Despite its computational origin, starting bloc selection is largely independent of specialized assumptions. This ribodinucleotide route to inheritance may also have facilitated 5′–3′ chemical RNA replication.

## INTRODUCTION

### The environment

We will calculate the likely events in a landscape with partially activated 5′ nucleotides. Nucleotides in randomized amounts meet at random times, subsequently reacting in loci called pools. Such pools might be realized after surf sprays over an uneven icy surface (Yarus 2012) or after soaking of a porous mineral congenial to RNA. While minerals usually bind 83-mer RNA (Biondi et al. 2017), smaller nucleotides may be less susceptible, or may remain active even when bound.

### The premise

Evolution using sporadically available precursors furnished all molecular capabilities for a primordial biota. Essential molecular capabilities probably evolved separately; separation requires fewer improbable molecules and events than innovations that appear together. Though the origins of more than half of the amino acids, 2 nucleotides (nt) (Powner et al. 2009), and the polar bits of biolipids can be unified as related HCN and H_2_S chemistry, this requires separate partial reaction streams that ultimately pool (Patel et al. 2015). Following Woese (2002), other biological inventions were probably first separated, with individual successes joining by horizontal transfer before the advent of reliable inheritance. These ideas validate study of single evolutionary successes, in ensembles of minimized complexity, and later union. Here we apply these ideas to the encoding of a new chemical function.

### Ribodinucleotides can be a simplified gene product

Though ribozymes are celebrated for simplicity, they are too complex to be likely initial gene products (Yarus 2015). As one measure of complexity, 5′–3′ complementary nucleotide-by-nucleotide replication requires large selected catalytic RNAs, over 200 nt long (Attwater et al. 2013). Accordingly, simpler, more readily synthesized ribonucleotide catalysts are better candidates for early roles. Notably, numerous small, chemically active RNAs exist, as dinucleotides and smaller (Yarus 2011). These are the coenzymes, many of which are universal, and therefore also ancient (White 1976; Yarus 2011). Moreover, many coenzymes are reactive even as peptide-free RNAs. Redox factors are a particularly prominent such class (Fischer et al. 2010), because more than 80% of modern protein redox enzymes gain their reactivity from such a bound small RNA (Puthenvedu et al. 2018). This suggests that a large portion of modern metabolism reflects the ancient existence of reactive 5′–5′ dinucleotides, with a structure similar to modern coenzymes (Yarus 2011). Therefore, here and elsewhere I assume that the first gene products were reactive ribodinucleotides.

### A simplified gene

For 5′–5′ dinucleotide reactions to be heritable, such molecules must be encoded by a transmissible agent. Novel non-Watson–Crick templating mechanisms provide several routes to encoded coenzyme-like RNAs (Puthenvedu et al. 2018). Thus, though this study initially sought self-complementary replication (Yarus 2012), cross-templating supplied a simpler, reproducible way of encoding 5′–5′ dinucleotides on uncomplicated linear homopolymeric 5′–3′ templates; e.g., AppA on poly (U) (Puthenvedu et al. 2015),

pA+ImpA⟶5′−3′poly(U)5′−5′AppA+Im

and GppG on poly (C) (Majerfeld et al. 2016).

pG+ImpG⟶5′−3′poly(C)5′−5′GppG+Im.

Para-templating is yet simpler, encoding AppA on GppG (Puthenvedu et al. 2018).

pA+ImpA⟶5′−5′GppG5′−5′AppA+Im,

where ImpA is 5′ AMP phosphate-activated by 2-methyl-imidazole (Im; Inoue and Orgel 1982). Thus our gene product resembles AppA, but one nucleobase, pairing like adenine, also is reactive (symbolized AppA*). For AppA*, complementary geochemically produced linear homopolymers can serve as an early gene. Such genes can be readily produced from activated nucleotides in the presence of montmorillonite clays (Ferris and Ertem 1992). This somewhat resembles Cairns-Smith's (Cairns-Smith 1982) suggestion of clay ancestry for nucleic acid information, but crucially, RNA gains only length (Ferris et al. 1996) rather than sequence information, from a preexisting mineral.

Alternatively, chemically produced GppG template emerges from solution reactions of activated pG (Kanavarioti 1997). Thus para-templating can occur in a pool that receives only activated and unactivated mononucleotides. Dinucleotide formation from nucleotides interacting in solution exists, but is slower. This solution reaction can be exploited by para-templating to produce GppG template. Accordingly, capturing either an environmental polypyrimidine, partially activated pG, or GppG, will supply a template for a coenzyme-like product, thereby encoding a chemical capability.

These gene-product systems do not replicate per se, instead relying on multiple gene-like molecules to convey inheritance. I argue (Yarus 2017) that this is not a bug, but a feature: selection for the complex function of 5′–3′ replication seems unlikely unless expression preexists to convey diverse advantages to entities possessing multiple gene-like molecules (but compare Chen et al. 2004). Accordingly, gene expression logically predates, is required for, and possibly initiates the evolution of 5′–3′ replication. This topic reappears in the Discussion.

### Simplified selections with differing intensity

In order to calculate selection effects, previously defined (Yarus 2017) mild and strong selections are applied to pools of varied ages. Selection probabilities increase linearly with pool concentration of the favored coenzyme-like molecule:

$$\text{mild}\;\text{selection}:P_{select}=0.25+0.5C_{rel},$$

$$\text{strong}\;\text{selection}:P_{select}=C_{rel}.$$

Strong selection makes the probability of pool selection/survival (*P*_*select*_) twice as responsive to the relative AppA*concentration, compared across pools (*C*_*rel*_), though mild and strong selections have the same mean probability of one-half. A distribution of product concentrations is calculated from integrated pool kinetics at a given age or age distribution (see Materials and Methods). Using mild and strong probabilities, individual pools survive according to their product concentrations. This yields new, selected pool populations with calculable properties (see Materials and Methods). Further details exist where these selections were defined (Yarus 2017).

### New properties in present calculations

Three new complications appear here (see Supplemental Information), each of which introduces a plausible effect, but which potentially hamper selection of heritable function. Thus, whether evolution of inheritance can occur is newly relevant.

### An unstable product

Previously the coenzyme-like product had the high observed stability of normal 5′–5′ dinucleotides (Yarus 2017). Instead, here selected AppA* is moderately unstable, consistent with potential reactivity of the pA* nucleotide, which might underlie its selected chemistry or physics.

### Multiple products

Previously (Yarus 2017) it was assumed that synthesis of a pooled AppA dimer was the unique, sufficient, selected goal. However, if partially activated pA, pG, and pA* are present, AppA, GppG, A*ppA*, AppA*, GppA*, and GppA are all made in present para-templating pools. However, by assumption here, only a single product, AppA*, is selected for function.

### Slower reactions

Previously, the fastest experimental synthesis, cross-templating of GppG, was modeled. Here two slower reactions with AppA-like products, are used instead, in order to model descent to modern A-containing coenzymes (Puthenvedu et al. 2018).

### Relation to recent progress in chemically activated templated 5′–3′ RNA synthesis

Imidazolide-activated nucleotides can also be incubated with a base paired primer on a template, thereby extending the primer at its 3′ end with accurately paired nucleotides. Such simplified, primed systems have added much to knowledge of templated, nucleotide-by-nucleotide 5′–3′ chain extension. For example, 2-amino-imidazole activation appears sevenfold better for extension (Li et al. 2017) than the more usual activating group, 2-methyl-imidazole (Inoue and Orgel 1982), that we also use.

Recent use of such a system shows that the immediate precursor in such a 5′–3′ linked extension is not the imidazolide-activated nucleotide itself, but a dimer formed from 2 nt imidazolides (Walton and Szostak 2016). Such imidazole-linked dimers, formed during incubation, are highly reactive and unstable (Walton and Szostak 2017), and accordingly facilitate 5′–3′ chain extension. We wish to relate this finding to present experiments.

### 5′–5′ dinucleotide products are a distinct case

However, there need be no relation. Dinucleotide product is quite different, having 5′–5′ connectivity and therefore resulting from 5′ attack on a 5′ phosphate (Puthenvedu et al. 2018). In fact, the RNA products above, with 2′–5′ and 3′–5′ linked ribonucleotides, are not easily detected in our incubations (Puthenvedu et al. 2015, 2018; Majerfeld et al. 2016). Thus, prominent imidazole-linked dimer-enhanced reactions must be much less frequent than those leading to 5′–5′ dinucleotide products.

Moreover, our reactions used 2-methyl-imidazole-activated nucleotides, and the corresponding activated dimer intermediate decays 26-fold faster than the 2-amino-imidazole dimer (Walton and Szostak 2017). The net result is that, even in 24 mM 2-MeImpC, a nucleotide concentration higher than our usual one, the 2-methylated dimer intermediate cannot be detected (Supplemental Material; Walton and Szostak 2017).

### Response to activated nucleotide

Thus, both product and precursor differ in this work. In addition, imidazolide-bridged dinucleotides are unstable and formed by reaction between two imidazolide-linked precursors. This suggests that in our days-long reactions, such activated dinucleotides would form in a rough steady state between second-order synthesis and a first order decay. In fact, the expected second-order synthesis and first-order decay have been confirmed experimentally (Walton and Szostak 2017). Thus, simplifying to the reactive species:

$${\left[N^{*}N\right]}_{ss}\approx \frac{k_{2nd}}{k_{d_{N*N}}}{\left[\text{ImpN}\right]}^{2},$$

where [N*N]_ss_ is the steady state concentration of reactive dimer N*N, *k*_dN*N_ is its first order decay rate constant, and *k*_*2nd*_ is the second order rate of such dimer synthesis. Dimer dependent synthesis should increase in rate with the square of the activated nucleotide, [ImpN]. But this is not observed, either for AppA cross-templated by poly (U) (Puthenvedu et al. 2015), GppG cross-templated by poly (C) (Majerfeld et al. 2016), or AppA para-templated by GppG (Puthenvedu et al. 2018). All are instead first order in activated and normal nucleotides. Therefore, cross- and para-templating, unlike 5′–3′ chain extension on a primed template, probably result from reaction of an activated (ImpN) and unactivated (pN) nucleotide.

### Representation of results

Evolution was previously evaluated using integrated AppA* synthesis via a templated route (temp) versus integrated synthesis by chemical encounter of activated and normal nucleotides in solution (chem). Their ratio, temp/chem, accurately embodies pool preference for templating over the widest range (Yarus 2017). However, for discussion of evolutionary progress, the fraction of total synthesis via templating (*f*_temp_) seems clearer:

$$f_{\mathrm{temp}}=\frac{\text{temp}}{\left(\text{temp}+\text{chem}\right)}.$$

Below, to focus closely on selection for encoded or templated synthesis, such data are extended to yield Δ*f*_temp_, mean pool increase in the integrated fraction of templated synthesis, after one selection.

$$\Delta f_{\mathrm{temp}\;}=f_{\mathrm{temp},\mathrm{after}\;\mathrm{selection}}-f_{\mathrm{temp},\mathrm{before}\;\mathrm{selection}}.$$

A population of pools survives to a given age; then, one by one, continues according to the probability of selection (*P*_select_) corresponding to active dinucleotide content (Simplified selection, above; see also Materials and Methods). Δ*f*_temp_ quantifies population progress toward complete encoding (*f*_temp_ ≡ 1) after this cycle of selection. Put another way, Δ*f*_temp_ quantifies selection of pools with histories that favor templated AppA* synthesis, rather than via stacked free nucleotides (Puthenvedu et al. 2015). Accordingly, Δ*f*_temp_ is the intrinsic selectability of templating, or is the minimal increase in templating (always < a heterogeneous molecular pool [Yarus 2017]) under selection for an encoded product. Δ*f*_temp_ ranges from 0 to 1; larger values imply greater selected progress toward templated synthesis. Surprisingly, one plausible selection can shift minority pool templating to predominantly encoded AppA* synthesis (Δ*f*_temp_ > 0.5).

## RESULTS

### The eighty routes

#### Pool life cycle

We consider populations of pools under “constant” danger (as from precipitation), which expire with overall exponential probability in time. These are contrasted with pools in “periodic” danger (as from tides), that expire at fixed intervals.

#### Pool age

“Early” (recently established) and “late” pools can show very different responses to selection (Yarus 2017), so pools of varied age are compared.

#### Selection intensity

We compare pools under “mild” and “strong” selection (Yarus 2017).

#### Nucleotide concentrations

**FIGURE 1.**
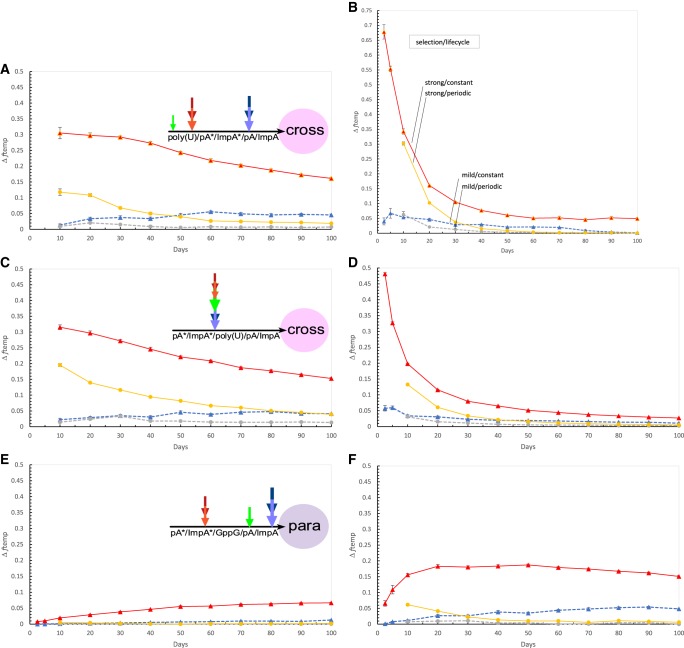

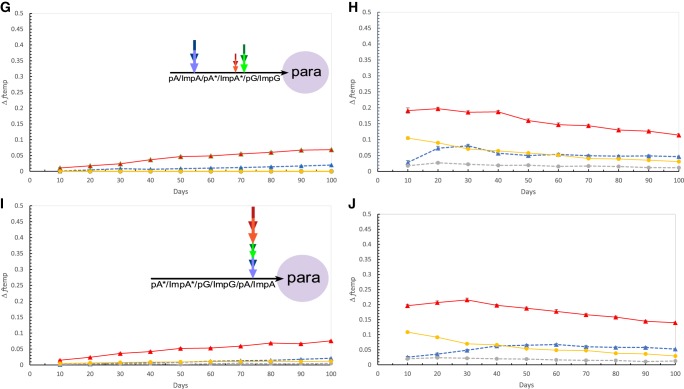
Fractional progress toward AppA* templating in one cycle of selection (Δ*f*_temp_) versus pool age. Solid lines indicate strong selection; dashed lines mild selection. Triangles are pools in constant danger; circles are pools in periodic danger. Means of ten simulations on 1000 pools are plotted; bars *around* points are standard errors (where invisible, errors are within the mean points). *Leftward* panels in each pair have a scheme at *upper right* showing a set of schematic substrate arrivals (random *downward* arrows) on a 10-d time axis (black *rightward* arrow). Relevant substrates are listed *beneath* the timeline. Pools labeled “cross” perform cross-templated synthesis; “para” implies para-templated synthesis. (*A*) Sporadically fed, cross-templated pool, 1 mM substrates. (*B*) 10 mM substrates. (*C*) Simultaneously fed, cross-templated pool, 1 mM substrates. (*D*) 10 mM substrates. (*E*) Sporadically fed, para-templated pool, 1 mM substrates. (*F*) 10 mM; GppG input. (*G*) Sporadically fed, para-templated pool, 10 mM substrates. (*H*) 100 mM; pG/ImpG input. (*I*) Simultaneously fed, para-templated pool, 10 mM substrates. (*J*) 100 mM; pG/ImpG input.

Substrate concentrations alter the way selection works, but also set a lower limit for geochemical nucleotide supply. In Figure 1, a “minimal” (on the left) and a higher, more “functional” (on the right) concentration are compared. All nucleotide concentrations vary together, so that mean concentrations can be characterized by one number.

#### Expression chemistry

We compare pools that make their selected product, AppA*, by “cross-templating” (Puthenvedu et al. 2015) or pools that proceed by “para-templating” (template GppG and product backbones identical [Puthenvedu et al. 2018]).

#### Nucleotide arrival

Pools can receive uncorrelated supplies of different nucleotides at random times and in randomized amounts; these are termed sporadically fed pools (Yarus 2012). Alternatively, nucleotide substrates can appear at random times and in random amounts, but arrive together from a single geochemical source (Puthenvedu et al. 2018). A para-templating pool has an additional degree of freedom; it can get environmental GppG template, or synthesize it internally from pool nucleotides.

For clear summary (as for the title), the routes are simplified as tests of the extremes of the six conditions above. Additionally, para-templating pools obtain GppG template externally or make it internally. So, there are 80 routes (taking the termini of lines in Fig. 1 as “routes”)—comparing outcomes for differing life cycles, pool ages, selection intensities, substrate concentrations, two para-templated syntheses, plus cross-templating RNA synthesis, and sporadic and synchronized random nucleotide supplies. Pool fates are calculated by integrating a route using measured or plausible rates and stabilities; that is, using measurements locally and from the literature (Yarus 2012, 2017). Agreeably, quantitating selection (Δ*f*_temp_) identifies a favored route for evolution of gene-like encoding.

#### Selection of templated product via 80 routes

Figure 1 summarizes selection. In 10 panels, each of five pairs of side-by-side graphs presents data for one environmental nucleotide supply (simultaneous or sporadic) and one RNA synthesis (cross- or para-templated). Each side-by-side pair of panels also has selection data for two substrate concentrations. There is a low mean nucleotide concentration (leftward; a mean of 1 mM cross-templated or 10 mM para-templated), yielding minimal function. In the second, rightward, panel populations are supplied with 10-fold higher mean nucleotide (10 mM cross-templated or 100 mM para-templated). Concentrations are chosen so pools can be compared when functioning similarly (compare right panels, or compare left panels); or alternatively, compared with identical nucleotide inputs (compare 10 mM nucleotides in all cases).

Each leftward paired panel has a scheme at upper right which symbolizes varying substrates (downward arrows of different sizes) arriving simultaneously (superposed arrows of varying size at random on the black time arrow) or sporadically (separated, varied arrows each at random on the time arrow), and whether AppA* synthesis is cross- or para- templating. Superposed arrows with related colors represent concurrent activated and unactivated nucleotides, whose levels are assumed equal.

Within each panel, populations of pools of varied ages in days (*x-*axis) are associated with their selected fractional progress toward templated synthesis (Δ*f*_temp_, *y*-axis) during one cycle of mild (dashed lines) or strong (solid lines) selection for active dimer. Further, distinct data appear in every panel for pools under a constant danger (triangles) or periodic danger (circles).

#### Extent of selection for templating

There are potentially as many conclusions as pairs of points, but we attempt to stress broad trends. The least resolved conclusion is that routes vary greatly in susceptibility to selection. Selection is effective (Fig. 1B,D), moderate (Fig. 1A,C,F,H,J) and minimal or nonexistent (Fig. 1E,G,I). Cross-templated synthesis can become a quick majority, in one cycle (e.g., Fig. 1B), while para-templating can be unselected at any age (e.g., Fig. 1G).

#### Pool life cycle

Almost without exception, pools with varied lifetimes (triangles, constant danger) are more affected by selection than are paired and matched pools with defined lifetimes (circles, periodic danger). Even apparent “exceptions” are (Fig. 1) very young pools under weak selection, where the two selections are too similar to be clearly distinguished. Later for these same pools, constant danger pools are superior to periodic danger at all pool ages.

Figure 1B,D and F show selection extended to very early times to emphasize maximal observable selection. Early cross-templating pools are unique. Very extensive template selection and strong improvement under intensified selection are characteristic (e.g., Fig. 1B). Quantitation is worth emphasis: all early cross-templating pools would become fully templating after a few cycles of selection, and mean strongly selected, cross-templating pools supplied with 10 mM nucleotides become majority templaters after one selective cycle (Δ*f*_temp_ ≈ 0.7). Even at 1 mM nucleotides on average (Fig. 1A), only a few cycles would select pools with templated AppA* synthesis. However, such selection requires a longer time, especially with mild selection, than at higher nucleotide concentration. The existence of a plausible pool history in which one selective cycle necessarily produces majority templating confirms the similar finding in a simpler, less restricted early pool (Yarus 2017). These data also confirm (Yarus 2017) substantial cross-templating selection at mean nucleotide concentrations ≥1 mM (Fig. 1A,C).

#### Pool age

In seven of ten panels, particularly under strong selection, earlier pools are more productive under selection. The superiority of early sporadically fed cross-templating pools under strong selection has been observed before (Yarus 2017). But early superiority is less true for para-templating ribonucleotides, and never true at all for mild selection on para-templating. Mild selection of sporadically supplied para-templating pools is the extreme case (Fig. 1E,G), where selection for product is negligible at any pool age. The superiority of recently established pools is explained below.

#### Nucleotide concentration

The most effective selection occurs at increased substrate concentration in all cases. Para-templating with internal template synthesis (Fig. 1G–J) requires that nucleotide inputs be extrapolated to high concentration (0.1 M) in order to support significant selection.

#### Selection intensity

Strong selection (solid lines) is particularly effective, and mild selection (dashed lines) particularly ineffective in the earliest pools in all cases. The strong/mild product ratio declines as a pool matures. This is true even at lower nucleotide concentration in para-templating pools (Fig. 1E,G), where small plotted values are difficult to read. Much greater response to strong than mild selection, earlier termed “disproportionate response to selection” (Yarus 2017), is confirmed in a broader pool context here, and explained in the next section.

#### Expression chemistry

Present selections mandate revision of earlier discussion. I previously suggested that para-templating (panels 1E–J) needs only one type of chemistry to create both 5′–5′ template and 5′–5′ product backbones. Its nucleobases are both purines, and thus potentially derived from one geosynthesis event (Oro and Kimball 1961). Para-templated expression might therefore be simpler and arise earlier than cross-templated ribodinucleotide synthesis (Puthenvedu et al. 2018).

But calculation of susceptibilities to selection, Δ*f*_temp_ challenges this argument. Para-templating pools (Fig. 1E–J) are less responsive to selection than comparable cross-templating pools (Fig. 1A–D). This can be divided into two effects. In Figure 1E,F, para-templating synthesis is evoked by environmental GppG template. This reduces and delays templating, particularly early templating, compared to optimal pools (Fig. 1A,B). A further deficiency appears if para-templating pools are asked to make template internally: now (Fig. 1G–J) roughly 10-fold more substrate is required to establish an encoded function. Moreover, selection of para-templated product is usually both delayed and requires elevated nucleotide (whether GppG synthesis is internal [at 100 mM pG] or external [at 10 mM GppG]; Fig. 1E–J).

Late pools receiving 100 mM nucleotides (compare Fig. 1B,D,F,H) are a special case. Such concentrated reactions are not impossible; the least soluble normal nucleotide, pG, is soluble to 0.59 M at 25° (O'Neil 2006). Thus, one reaction to these calculations would be experimental exploration of para-templating in nucleotide slurries. However, even at high nucleotide concentrations, selection does not work optimally until 20–60 d (extrapolated using rates from low concentrations, assuming ideal behavior at high concentrations; Fig. 1F,G). Even the most favored para-templating is likely delayed with respect to cross-templating (Fig. 1B,D versus Fig. 1F,G).

Thus, cross-templating pools evolve rapidly at early times (Fig. 1A–D) when contemporaneous para-templating ones in similar environments would be near-quiescent (Fig. 1E–H). So para-templating is difficult to select, requires high normal and activated nucleotide concentrations, and is likely to cycle through selection slowly. Unless other para-templating reactions with more favorable behavior are discovered, cross-templating pools seem more proficient in a primordial setting with likely low nucleotide concentrations. However, search for new para-templating reactions would be reasonable: its geographic advantage is untouched by these present arguments—it is still easier to imagine its nucleotides appearing in one locale.

#### Nucleotide supply mode

In contrast to Expression chemistry immediately above, the differences between sporadic (random independent) arrival for all nucleotides and simultaneous random arrival, perhaps from a unified source, are minimal. This is true throughout relevant pairs for all routes, as can be seen by comparing Figure 1A and C, and/or Figure 1B and D, and/or Figure 1E and G, and/or Figure 1F and H. Though intuition might suggest otherwise, the stability of nucleotides and intermediates on pool time scales of months (like GppG during para-templating) make it effectively unimportant on what schedule stable substrates arrive. This is related to the advantage derived from accumulation of stable nucleotides in sporadically fed pools (Yarus 2013), and can be restated as an advantage of pooled synthesis. Pools (Fig. 1) are broadly insensitive to different relative substrate arrival schedules.

Para-templating with external GppG supply (Fig. 1E,F) is superior to internal pool synthesis of GppG from partially activated pG (Fig. 1G–I), because higher nucleotide concentrations are required for template synthesis in the latter case.

These varied outcomes are diverse results of a simple underlying mechanism, to which we now turn.

### The starting bloc selection

#### Separating selection into dispersion and stringency

Understanding distinctions among 80 routes (Fig. 1) requires treatment of the functional nature of selection (see Materials and Methods). In particular, in a varied environment, selection depends on the shape of distributions. To simultaneously clarify effective selection, discussion below also uses near-optimal pools.

**FIGURE 2.**
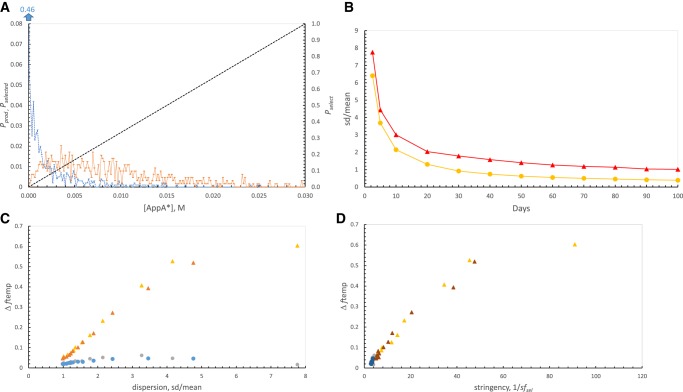
The functional nature of selection. (*A*) Selection for constant danger pool product concentrations. Mean pool age is 20 d, with 5 mM mean nucleotide supplies. Probabilities are shown before (blue dashed, *P*_*prod*_) and after (ochre solid, *P*_*selected*_) a cycle of strong selection (black dashed, *P*_select_, *right y*-axis). Normalized probabilities are calculated from 1000 pools (unselected) and 642 pools (after selection). The first bin is 10^−10^ M wide, all other bins are 1.5 × 10^−4^ M. Blue arrow at *top left* points to inactive pools (0.000 M product). (*B*) Product dispersion versus pool age. The same mean age and nucleotide concentration as in *C* and *D* are used. Triangles are pools in constant danger, circles pools in periodic danger. (*C*) Pool progress toward templating (Δ*f*_temp_) at differing product dispersions (SD/mean). Using 20 d, 5 mM pools as A, Δ*f*_temp_ is plotted versus initial, unselected pool product dispersion (SD/mean). Triangles are strongly selected constant danger pools (sporadically and simultaneously fed). Circles are mildly selected constant danger pools (sporadically and simultaneously fed). Points are ordered (Fig. 2*B*), early pools on the *right*, late to the *left* (see text). (*D*) Pool progress toward templating (Δ*f*_temp_) at differing stringencies (1/*sf*_*sel*_). Using 20 d, 5 mM pools as *A*, Δ*f*_temp_ is plotted versus selection stringency (1/*sf*_*sel*_). Triangles are strongly selected constant danger pools (sporadically and simultaneously fed). Circles are mildly selected constant danger pools (sporadically and simultaneously fed).

Figure 2A contains product distributions (*P*_*prod*_; plots of probability versus product concentration) for a cross-templating pool population in constant danger, fed sporadically with 5 mM nucleotides, at a mean pool lifetime of 20 d. The unselected population (dashed line) carries out 59% of dimer synthesis via templating. At 20 d mean lifetime, many unselected pools have not begun synthesis: 46% of all pools are unproductive (blue arrow, Fig. 2A). This population undergoes strong product selection; the diagonal dotted line plots selection probability (*P*_*select*_) on the rightward *y-*axis.

A new normalized distribution is selected (ochre solid; *P*_*selected*_). It is depressed by unlikely selection at low product (leftward) and consequent low *P*_*select*_, and enhanced at high product (rightward) and high *P*_*select*_. Selection increases average product from 1.6 mM unselected to 10.8 mM after selection, as expected for a favored AppA* molecule. Selection can be described by three functions of product concentration:

$$P_{selected}=\;P_{select}*P_{\;prod}.$$

We know *P*_*select*_ (see Simplified selection, above, Fig. 2A) and have determined *P*_*prod*_ by kinetic calculations for numerous pools (Fig. 2A, dashed; see also Materials and Methods). A crucial idea is that *P*_*selected*_ is determined by the initial shapes of *P*_*prod*_ and *P*_*select*_. If initial *P*_*prod*_ is a sharp, symmetrical peak, strong selection will have little effect. If initial *P*_*prod*_ is broad (product concentration is disperse), pools with high product concentration dominate the outcome by multiplying larger *P*_*select*_, shifting *P*_*selected*_ upward, producing effective selection.

#### Predicting selection

Accordingly, an index for initial *P*_*prod*_ shape should rationalize the success of selection. Starting with such an index of *P*_*prod*_ dispersion, we expect that an even broader rationalization of selection will result from an index that carries information on *P*_select_*P_*prod*_ (thus reflecting both stringency and dispersion). In fact, *P*_select_*P_*prod*_, integrated, is the probability of pool survival after selection (*sf*_*sel*_).

So, pool selection follows (standard deviation/mean) as an index for *P*_*prod*_ dispersion. (SD/mean) is a standard statistical measure of dispersion sometimes called the coefficient of variation (Sokal and Rohlf 1995). Moreover, selection also more broadly follows the reciprocal of fraction surviving selection (stringency; 1/*sf*_*sel*_), an index related to 1/(*P*_select_*P_*prod*_). Especially used together, dispersion and stringency allow concise explanation of successful selection in terms of observable pool properties.

#### Dispersion and selection are a function of age

Figure 2B shows dispersion (SD/mean) of constant- and periodic-danger cross-templating pools—both dispersions increase sharply at early times. Highly selectable early pools of Figure 1B, both constant and periodic danger, are therefore specifically the highly disperse ones. Decreased selection as pools age (Fig. 1B) occurs because later pools are less disperse (Fig. 2B), but secondarily because late pools can approach complete unselected templating and so lack scope for selected increase (Yarus 2017). Figure 2B hints at more complete discussion below, showing that superior selection in constant danger at all ages (red triangles) occurs because pools are always more disperse than in comparable periodic danger (yellow circles).

#### Templating is a function of pool product dispersion, SD/mean

In Figure 2C, fractional selected progress toward templating, Δ*f*_temp_, is plotted versus the initial dispersion of product concentration. These 5 mM substrate data share with similar previous cases (Fig. 1B; Yarus 2017) maximum Δ*f*_temp_ > 0.5 at large SD/mean—that is, stringent selection in a disperse population produces majority templating after one selection. Two sets of data, for sporadic and simultaneous substrates, are combined; these have similar product distributions—as noted above, relative timing of substrate arrival is not an influential variable. In fact, mild (circles) and strong (triangles) selections each form a coherent group, with coherent internal relations between Δ*f*_temp_ and SD/mean. Moreover, points in Figure 2C are ordered in time: the earliest pools are on the right. Successively less-well-selected pools to the left are later, and also less variable (Fig. 2B).

Thus, we can accurately predict selection from relative pool dispersion (SD/mean) without population calculations. Moreover, we can posit a less explicit rule-of-thumb; more dispersion (with similar distributions), better selection (Fig. 2B). The earliest pools are an exception, treated in the aside below.

#### Selection is a yet more general function of fraction of pools surviving selection

In Figure 2D, fractional selected progress toward templating, Δ*f*_temp_, is shown versus stringency, 1/*sf*_*sel*_. While the data of Figure 2C are replotted, the plot is now notably more ordered. Selection for templating, Δ*f*_temp_, increases proportionate to 1/*sf*_*sel*_, and differing substrate supply, selection intensities and pool ages are unified. Thus stringency (1/*sf*_*sel*_, related to 1[*P*_select_*P_*prod*_]) more broadly rationalizes selection; our knowledge of selection in yet unseen populations is strengthened. Scatter in these plots is mostly due to the stochastic behavior of underlying pools, with smaller local digressions due to a limit on selection described just below. As expected, the unity in Figure 2C and D is not observed if different product distributions, as for constant and periodic danger pools (Yarus 2017), are plotted together.

#### Starting bloc selection acts on inevitable pool variation

**FIGURE 3.**
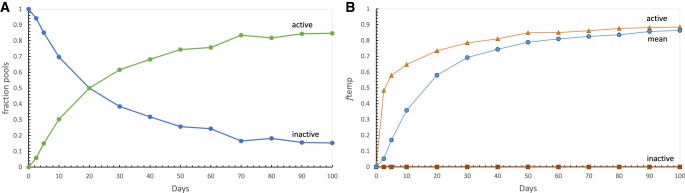
Division into active and inactive pools creates a selection opportunity. (*A*) Pool populations consist of active and persistent inactive pools. Fractions of 1000 sporadically fed cross-templating pools receiving 5 mM nucleotides that have made product (green circles; active) and not made product yet (blue circles; inactive) are plotted versus pool age in days. (*B*) Fraction templated synthesis (*f*_temp_) in 1000 active, average, and inactive pools from *A*, plotted versus pool age in days.

We can now define starting bloc selection. Figure 3A shows variation inevitably accompanying establishment of a population of sporadically fed pools. The fraction of pools that have begun making product, or still without AppA* product, are plotted versus mean age. Importantly, quiescent pools are a persistent class. In Figure 3B, the mean fraction templated product synthesis, *f*_temp_, is shown for mean, active, and still inactive populations of Figure 3A. Early pools that by chance get all substrates quickly and begin product synthesis, are a favored subset with an especially large templated fraction, especially quickly produced. Thus, early selection for pools with active product necessarily chooses between a majority which have done nothing (Fig. 3A), and a small minority that use templating more than 10-fold more frequently than average (2.5 and 5 d, Fig. 3B). This disperse distribution is ideally suited to selection (as in Fig. 2C) and also benefits from complete elimination (Fig. 2A) of still-inactive pools (elevated 1/*sf*_*sel*_, Fig. 2D). Accordingly, starting bloc selection can radically boost the population's templated synthesis of a selected product (increase Δ*f*_temp_). After a brief aside, parallel reasoning will largely explain overall selection results (Fig. 1).

#### An aside about very early times

**FIGURE 4.**
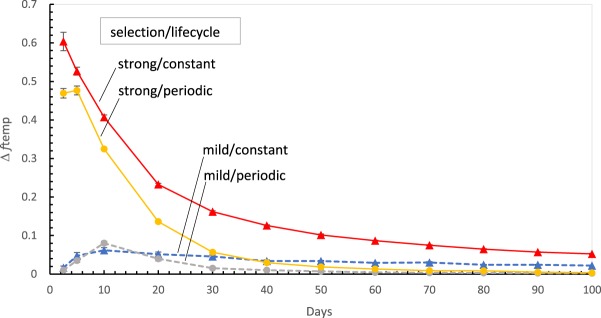
Selection (Δ*f*_temp_) is limited in very early pools. Sporadically fed cross-templating pools getting 5 mM nucleotide substrates on average are utilized. Legend is identical to panels of Figure 1.

Another effect alters selection at very early times, and decreases selection for early pools in global plots like Figure 2C and D. This effect is evident in Figure 4, a panel like those of Figure 1, but for the current example (Figs. 2, 3): 5 mM mean nucleotide input, and sporadically fed, cross-templating pools. Three of four Figure 4 selections can be seen to be less effective in very early pools: only strongly selected, constant danger pools appear to improve smoothly as pool life shortens. So, selection cannot be improved to *f*_temp_ = 1 by selecting more recently founded pools. Instead, an optimal selected improvement and time exists; Δ*f*_temp_ ≈ 0.07 for mild selection around 10 d and Δ*f*_temp_ ≈ 0.48 for strong selection at around 5 d for pools in periodic danger (Fig. 4).

Early selection decline exists because fractional templating (*f*_*temp*_) begins at zero (see also Materials and Methods). Persistence of more stable nucleotides in our current pool examples implies, early on, that mean chemically synthesized dinucleotide product increases as pool age^2^, while mean early templated product increases as age^3^ (Yarus 2017). Thus the ratio of templated to chemical synthesis increases linearly at early pool ages, beginning at zero (Yarus 2017). Because the productivity of template catalysis is selected, early pool populations with very low template levels are unproductive; early synthesis is largely chemical. In this early limit, templating can even be impossible (if a pool has no template yet). Once templating is well-launched, strong selection begins its decline due to decreasing population dispersion (Fig. 2B). Thus, there is an intermediate, but early, selection optimum (Fig. 4). Selection's reach for rare templating extends lower with more intense selection, so the optimum appears earlier for strongly selected than for mildly selected pools (Fig. 4). Efficient selection under constant danger (Fig. 1A–J) places the optimum off-scale at top left in Figure 4 only for near-optimal, strongly selected, constant danger pool populations.

#### All selection for templating can be rationalized

Starting bloc selection accounts for relative pool progress toward heritable ribonucleotide expression along the 80 routes (Fig. 1).

#### Pool life cycle

Pools in low, ≈ constant danger (as from meteors) yield superior selection almost uniformly in these studies, relative to populations at periodic danger (as from sunlight), when populations are matched for mean age. This is somewhat surprising, because constant danger produces frequent pools that perish early and so remain barren (Fig. 2A, see also distributions in Yarus 2017). But crucially, a constant danger population has varied ages, particularly a minority of longer-surviving pools with abundant templated product (Yarus 2017). This long-lived minority increases dispersion and facilitates selection because its elevated product is selected with high probability. Selection is therefore a matter of distributions (above, Fig. 2; below, Discussion).

#### Pool age

The clear superiority of early (recently established) pools under selection, except for some para-templating cases (Fig. 1), is explained by Figure 2B, which shows the rapid decrease of unselected relative variation, SD/mean, as early pools age. This behavior in turn is accounted for in Figure 3A, which shows how the division of the population into inactive and active pools wanes with time. Later pools accordingly converge to the population mean (Fig. 3B), and starting bloc selection becomes less and less productive. Thus the previously discussed “optimal pool succession” (Yarus 2017), in which selection succeeds early and selected pools later make abundant product, is specific to rapidly evolving cross-templating pools (Fig. 1A–D).

#### Selection intensity

Increasingly effective selection (increasing Δ*f*_temp_) depends on starting bloc selection (Fig. 3B). The greater the distinction between product levels (the greater the slope of *P*_*select*_ with product concentration; Simplified selection, above), the greater is the improvement in the ultimately selected population. As defined here, strong selection also removes inactive pools, which greatly improves selection's result (Figs. 2A, 3B). Together, these effects produce disproportionate increase in templating under strong selection (Fig. 1; Yarus 2017).

#### Mean nucleotide concentration

Greater mean nucleotide input, on average, implies more dispersion because, in the sporadically fed pool, substrate input varies from zero to a maximum related to spike size. Thus, product dispersion is greater with larger substrate spikes (Fig. 1).

#### Expression chemistry

Para-templating is almost universally inferior to cross-templating under selection (Fig. 1) because it obstructs starting bloc selection, in two ways. Firstly, para-templating can be implemented so as to require only nucleotides. Such pools perform internal synthesis of template, utilizing untemplated synthesis of GppG (Puthenvedu et al. 2018), presumably from stacked activated and unactivated nucleotides (Puthenvedu et al. 2015; Majerfeld et al. 2016). However, para-templating pools cannot profit from this added proficiency because template synthesis is delayed, and product selection is delayed until template accumulates. Given that para-templating has a smaller advantage in rate over chemical synthesis from the outset (Puthenvedu et al. 2018), it requires both high nucleotide concentrations and delays selection (Fig. 1G–J).

But even para-templating implemented to receive presynthesized GppG is hindered by its lower templating velocity (Puthenvedu et al. 2018), relative to cross-templated examples. This independently curtails product level in the starting bloc, and therefore interferes with its selection.

#### Nucleotide supply mode

Sporadic nucleotide supply is somewhat better than simultaneous supply (Fig. 1), because more varied reactions generate somewhat more population dispersion. However, the differences are small because of excellent survival of stable substrate and intermediate ribonucleotides on the time scale of these pools. In fact, a striking previous finding (Yarus 2017) is confirmed: templated gene expression may be selected only days after required nucleotides meet (Fig. 1).

#### Average, frequent properties are discussed

Statistical discussion should not suggest that evolution depends on rare success. The reverse is true. A large fraction of a favorably selected population uses templating (Figs. 1, 4, Materials and Methods). Subsequent evolution is based, not on unusual reactions, but on abundant templated synthesis.

## DISCUSSION

### Principal products

The predominant molecular products of reactions between 2-methyl-imidazolide activated nucleotides, incubated under varied solution conditions, are 5′–5′ dimers, resembling enzymatic cofactors (Puthenvedu et al. 2015, 2018; Majerfeld et al. 2016). Accordingly, we suppose that useful chemical or physical capabilities of active dinucleotides can be selected among such pool populations (Yarus 2011). This ultimately accounts for universal coenzymes in contemporary organisms (Puthenvedu et al. 2018).

### Selection of gene-like activity

Random meetings between arbitrary amounts of activated 5′ nucleotides, under selection, readily generate (Yarus 2017) encoded synthesis of active dinucleotide, rather than its uninstructed chemical synthesis. Thus, the structuring effect of selection on chemical pools (called chance utility [Yarus 2013, 2016]) is sufficient to produce inherited encoding of a new chemical capability. Simple genetic behavior emerges from a nongenetic predecessor.

Figure 1 evaluates previously observed influences on this kind of pool synthesis and selection. As an almost inevitable consequence of establishment of a population of selected pools, the early (not late), sporadically fed (not simultaneously fed), cross-templating (not para-templating) pool which receives several mM nucleotides while in constant danger (not periodic danger), under strong (less likely mild) selection for a coenzyme-like product evolves rapidly to possess an encoded product (Fig. 1). Such pools, active early (the starting bloc), possess qualities favorable to selection: high dispersion (Fig. 3A) and high stringency (Fig. 3B) naturally coexist. Using known reactions, a nascent starting bloc's elevated templating (Fig. 3B) mandates encoded synthesis after it is selected.

### Only ordinary chemical means are used

Notably, these calculations rely on experimental data on RNA reactions (or reactions comparable to these), and consequences attributable to ordinary chemical kinetics (though sometimes extrapolated to elevated concentrations; Figure 1H,J; Supplemental Information). Three novel conditions have been introduced without preventing the evolutionary transition. Multiple products are allowed, though only one will be selected (New properties, above). Smaller rate constants observed for AppA synthesis are used throughout (Puthenvedu et al. 2015, 2018). Selected AppA* product is unstable by hypothesis, with a mean lifetime of 100 d (*t*_½_ = 69 d). Thus alongside original conclusions (sporadic nucleotide supplies, realistic substrate decays, short pool lifetimes, millimolar nucleotide concentrations are not bars to evolution [Yarus 2017]), we add insensitivity to these new conditions. Thus the scope of chance utility (Yarus 2013, 2016) in a ribonucleotide pool is extended. Such results also make an evolutionary role for the sporadically fed cross-templating pool more probable, and more such results can ultimately confirm it beyond doubt (Yarus et al. 2005).

### The sporadically fed pool is indispensable

Three essentials for selection of templated synthesis are critically tied to a sporadically fed pool; that is, they do not exist in a conventional research reaction.

### Accumulation of pooled precursors

While imidazole-activated nucleotides are an exception, most reactants and products in these reactions persist across the pool timescale in Figure 1. This implies that mean sporadically fed, cross-templated synthesis will initiate its increase as (pool age)^3^ (Yarus 2017); for example, this trend implies robust templating in late pools (see Materials and Methods).

### Chance utility directs pool alterations

When nucleotide arrival and amounts vary, selection for product chooses pools whose random histories favored the selected product (Yarus 2016). For example, selection may choose pools whose random substrate supplies elevate reactant concentrations and arrive at near-ideal times (Yarus 2013). Resulting restriction to pools favorable with efficient reactions is termed chance utility (Yarus 2016). This choice can be permanent. For example, when pools possess multiple reactions, selection can eliminate an abundant inhibitor (Yarus 2016). Progressive chemical adaptation in pools therefore exists before genes exist. Most particularly, chance utility can select an encoded, rather than chemically formed product (Yarus 2017). This recalls Schrödinger's (Schrödinger 1944) remark that a living thing must eat a low entropy diet, in order that life not violate the Second Law. The current development shows that election of a similar low entropy result can initiate inheritance; because of template catalysis, selection naturally focuses on proto-biological behavior.

### Starting bloc selection exploits pool heterogeneity

Optimal selection is also intrinsically a pool event. Initiation of a pool population necessarily splits pools into inactive and active instances. As shown in Figures 2 and 3, selection applied during splitting inevitably acts on a highly disperse population (Fig. 3A) which, under strong selection, stringently yields only a small fraction of pools (Fig. 3B) containing individuals unusually proficient in templating. A selected starting bloc therefore accelerates evolution.

### A nonpool characteristic supplies an elevated synthetic rate: template catalysis

A final essential for selection of inheritance is not a pool characteristic, but a molecular one: template catalysis (Yarus 2017). A template can also be an entropic catalyst for bound complements (Yarus 2016), speeding reaction by conjoining them (Puthenvedu et al. 2015). Increased mutual reactivity between nucleotides on a template is the ultimate basis of selection for more product.

### Independence of specific assumptions

As argued previously for template catalysis (Yarus 2017), essential events in this route are not dependent on special model conditions. So: templates bring bound nucleotide reactants together, and can accelerate reactions without appealing to special arguments. Pool effects here are similar: pool accumulations come automatically from conservation of mass and its kinetic consequences (Yarus 2017). Chance utility seems inevitable when favorable pool reactions supply a selected product (Yarus 2013, 2016), and starting bloc selection is likely because sporadically fed pools begin useful activity at broadly varying times (Fig. 3). While this pathway was discovered by calculation, it is plausible independent of its specific derivation. To summarize: chance utility, acting via a preference for disperse, templated coenzyme-like dinucleotides, uses stringent starting bloc selection to quickly establish majority encoded/genetic expression.

### Rules-of-thumb for improved selection

Above, differences in selection are explained. But a reversed logic is also useful when a new selected outcome is sought via rational changes in mechanism. If seeking faster evolution, three tactics have been useful:

1. Increased dispersion of the distribution under selection (e.g., SD/mean of a favored product; Fig. 2B,C) will likely yield increased selection. This is demonstrated here for variations in pool life cycle, age (see Fig. 3B), nucleotide concentration, expression chemistry, and to a lesser extent, nucleotide supply mode. Selected starting bloc change is near-proportional to dispersion over a sevenfold range (Fig. 2C), except very early, where limited by low template activity (Fig. 4).
2. Utilize more intense selection; this stimulates change, independent of dispersion effects (Separating selection, above). Here, such stimulation acts on the preexisting product distribution in two reinforcing ways: larger product effects (*P*_*select*_/product concentration), and decreased acceptance of inactive pools (all panels, Figs. 1, 2A). Both independently increase the impact of an active pool minority (Fig. 3).
3. Changes that increase selection stringency (1/*sf*_*sel*_) for favored entities can combine effects 1 and 2 just above to enhance selection. Stringency here varied about 40-fold, with near-proportionate effects on starting bloc selection (Fig. 2D), except where limited by early template rarity. The starting bloc's importance depends on near-automatic coordination of tactics 1, 2, and 3 to elevate Δ*f*_temp_ (Figs. 1B, 4).

### Selection alters majority pool chemistry

**FIGURE 5.**
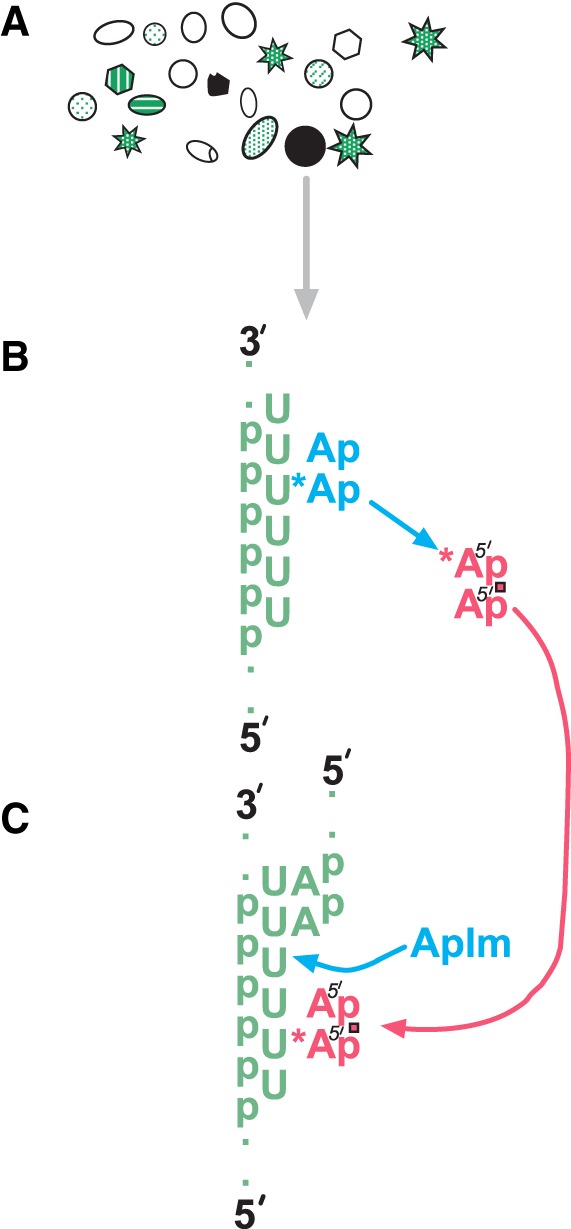
Descent of templating. This figure summarizes the route to inherited, proto-genetic characters. (*A*) Sporadically fed pools getting varying substrates; a minority makes elevated, useful product. (*B*) Selected synthesis of active AppA*, via the quantitatively favored cross-templated route. (*C*) Evolution of templated 5′–3′ RNA synthesis may be stimulated by 5′–5′ ribodinucleotides.

Figure 5 depicts starting bloc selection. Each small geometric feature in Figure 5A is a sporadically fed pool. Pools have varied forms and contents representing different histories (as in Fig. 1, including quiescent ones). Only a minority (Fig. 2D) offers elevated functional product, adjoining an arrow indicating selected descendants (whose biochemistry appears in Fig. 5B). The majority without descendants vary in content because there are multiple ways to be unproductive. For example, pools that have received only template, or only pG nucleotides related to template, differ internally but are all unproductive, with little or no AppA*.

A significant unproductive pool has a star-like outline, symbolizing para-templating pools. In Figure 5A, these are more numerous than cross-templating pools (Fig. 1A–D) because nucleotide requirements for para-templating may be more frequently satisfied (Puthenvedu et al. 2018). But para-templating pools are not easily selected, are slowly developing, and require ≈100-fold higher nucleotide concentrations (Fig. 1G–J). So, stellate pools (Fig. 5A) using known para-templating routes will likely not compete with highly selectable, quickly developing, cross-templating pools efficient with mM nucleotides (Fig. 1A,C).

### Consequences of selection for elevated ribodinucleotide

Figure 5B explicitly shows narrowed activity in a permanently changed population after chance utility. Synthesis of a coenzyme-like molecule is now focused on the more productive cross-templating route (Figs. 1A–D, 4) exploiting poly (U) template catalysis (Yarus 2017). An enhanced chemical reaction (of A*) or physical property (such as absorbance) of AppA* provides a selectable pool character. Evolution is pleasingly effective; selection increases templating (Figs. 1B, 4), but also ensures that increase is evolutionarily useful (Yarus 2017; Puthenvedu et al. 2018; see also Materials and Methods).

### A potential relation to 5′–3′ chemical RNA replication

Figure 5C sketches a more speculative relationship. Early expression may have facilitated 5′–3′ RNA replication, and thus onset of traditional Darwinian evolution. Vogel et al. (2005) found that complementary trinucleotides adjacent and downstream from the site for polymerization of a templated 2-methyl-ImpC accelerated C incorporation by 40%–80%. This is likely due in part to creation of a substrate pocket flanked by stabilizing stacking interactions (Fig. 5C) for the incoming templated nucleotide (Tam et al. 2017). Such paired downstream helpers, in fact, make possible the templated insertion of all four natural chemically activated 5′ nucleotides, even A and U (Deck et al. 2011). Helper enhancements are even greater, several-hundred-fold, if the downstream helper has a proximal activated terminus like a 2-methyl-imidazole-activated 5′ phosphate (Prywes et al. 2016). This stimulation, in turn, is likely due to the formation of an activated dimer involving the incoming templated nucleotide and the 5′ terminal nucleotide of the helper (Walton and Szostak 2016).

Thus, both noncovalent and covalent enhancement of 5′–3′ primed and templated chemical RNA synthesis exist, dependent on adjacent downstream paired nucleotides. Figure 5C suggests that varied 5′–5′ ribodinucleotides, arising by cross- and para-templated pathways considered here, be considered for this stimulatory role. Our investigations show that these could be varied in composition and present in relatively high concentrations; such 5′–5′ ribodinucleotides would likely pair readily with a 5′–3′ template strand (Puthenvedu et al. 2015). Varied 5′–5′ dinucleotides, prior to the era of sequential templated RNA replication, might therefore help evolve a chemical form of templated RNA synthesis by serving as transient downstream helpers (Fig. 5C).

## MATERIALS AND METHODS

### Models

Quantitative results are from numerical solution of systems of ordinary differential equations describing reaction kinetics in pools of para-templating (Puthenvedu et al. 2015) and cross-templating (Puthenvedu et al. 2018) nucleotides. Equations for random nucleotide supply, synthesis, decay and interconversion, as well as rate constants, may be found in near-standard kinetic notation in four Supplemental sections, where the full simulation code for sporadically fed cross-templating (I), simultaneously fed cross-templating (II), sporadically fed para-templating (III), and simultaneously fed para-templating systems (IV) is shown. Equations were numerically integrated using the 4th-order Runge-Kutta integrator of Berkeley Madonna v. 8.3.23.0, usually with an integration step of 0.001 d, running under Microsoft Windows 7 on a Lenovo T440s with 16 GB RAM. Primary integrated kinetic data were further analyzed after transfer to Microsoft Excel 2013.

### Calculation of the index for selected change

Major results rest on comparisons of populations of pools with different histories, often with pool behavior summarized by a numerical index for response to selection for the product AppA* (Δ*f*_temp_). “Response” means increase in the integrated fraction of total AppA* synthesis conducted on a template (*f*_temp_). Thus discussion invokes increase in templated synthesis as an indirect consequence of selection for increased product AppA*. The rest of Materials and Methods tries to make the origin of this key index intelligible.

### Synthesis in individual simultaneously supplied, para-templating pools

**FIGURE 6.**
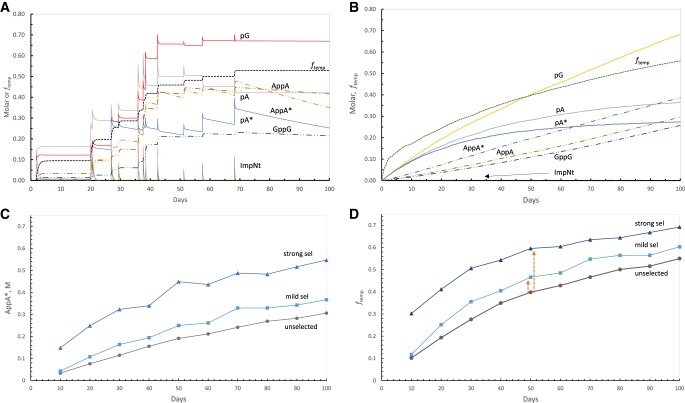
Calculation of selectability (Δ*f*_temp_) in simultaneously fed para-templating pools. (*A*) A representative pool history for a pool making GppG template internally, with labeled product concentrations. Note particularly stable nucleotides (e.g., pG), somewhat unstable nucleotides (e.g., AppA*), and unstable mixed activated nucleotides (e.g., ImpNt; 2-methyl-imidazolyl-5′-nucleotides). Note also nucleotide concentrations in pools receiving substrates randomly, but at a mean of 100 mM. (*B*) Means for 1000 pools like the one in *A*. Note particularly the integrated fraction of templated AppA* synthesis, *f*_*temp*_, which is zero at pool initiation. Thus there are necessarily early times when templating is negligible with respect to untemplated, chemical product synthesis. (*C*) Mild and strong selection of a functional ribodinucleotide product, AppA*. Data are means of 1000 pools. (*D*) Response of fractional templated synthesis (*f*_*temp*_) to selection for total product AppA*. The short dashed arrow is average Δ*f*_temp_ as a result of mild selection at 50 d; the longer dashed arrow is Δ*f*_temp_ as a result of strong selection at 50 d in 1000 pools. These quantities are also plotted in Figure 1J.

For this calculation of Δ*f*_temp_, (Fig. 6) data are from para-templating pools, chosen because their behavior is not previously published. Data for contrasting cross-templating pools have appeared (Yarus 2017) and appear here (Fig. 1A–D). Example calculations (Fig. 6A) use simultaneous random supply of nucleotides, pA, pA*, and pG (quantities distributed as Gaussians [≥0; mean magnitude 100 mM ± 50 mM {SD}], alongside equal quantities of imidazole-activated derivatives). Here, nucleotide supply probabilities always yield a mean of 1 nt arrival/10 d. Activated nucleotides (ImpNt), have the shortest pool lifetimes (e.g., ImpG: average 0.77 d, *t*_½_ = 0.53 d), so appear as spikes because they appear quickly, then decay in a few days. Normal nucleotides like pA and pG are extremely stable on this time scale (Yarus 2017), and so (though consumed in synthesis) persist and accumulate. Reactive nucleotides like pA* and AppA* have an intermediate stability, evident from their slower, but appreciable decay during Figure 6A. Notably, the internal synthesis of GppG template and the low rate constant for its synthesis mandate that G nucleotides must be supplied at high levels (*y-*axis, Fig. 6A) in order to support para-templated synthesis. Nonetheless, even though templated AppA* appears and can be selected (as documented in Fig. 1H), *f*_temp_ in Figure 6A shows that para-templated AppA* only becomes equal to chemically produced AppA* late in this unselected reaction.

### Synthesis in average simultaneously supplied, para-templating pools

For reliable generalization, mean pool behavior was calculated (Fig. 6B) by combining 1000 pools like the one in Figure 6A. Averaged 5′ nucleotides initially accumulate linearly, but fall below that level because of consumption and decay (particularly for pA*). Early dinucleotides like AppA* accumulate as (Days)^2^ if made chemically or (Days)^3^ if templated, or a mixture of the two (Yarus 2017). Templated products are therefore a minor fraction of the total at the earliest times. Short lived reactants like activated nucleotides, on average, decay and do not accumulate like stable ones, but are present at a mean of *k*_supply_/*k*_decay_ = 0.0077 M (after transient accumulation; neglecting consumption in synthesis [Yarus 2017]), and their presence can be seen just above the time axis in Figure 6B.

### Selection for the product, AppA*

Selection of AppA* is shown in Figure 6C, where gray circles are the means of 1000 unselected pools at 10 d intervals, blue squares are the average after mild selection (see Simplified selection, above), and triangles are the mean of a population created by strong selection. Clearly, selection for elevation of the active dinucleotide is effective throughout the life of averaged simultaneously supplied, para-templating pools.

### Templating after a selection for increased AppA*

Figure 6D shows the overall fraction of AppA* synthesis by template catalysis (*f*_temp_), for the same pool populations as Figure 6C. The short and long reddish arrows are therefore, respectively at 50 d, increase in fraction of synthesis templated, Δ*f*_temp_, for mild selection, and Δ*f*_temp_ for strong selection, as first indicated in Figure 1H. Inspection of the intervals between the lines in Figure 6D shows that strong selection has a similar effect throughout a 100 mM para-templating pool's lifetime. Thus for example, Figure 6D shows that para-templating pools are very unlike near-optimal sporadically fed, cross-templating 10 mM ribonucleotide pools (Fig. 1B) that sharply elevate selection at early times (Figs. 1B, 4).

## SUPPLEMENTAL MATERIAL

Supplemental material is available for this article.

## Supplementary Material

Supplemental Material
