# Supplementary material for: Eighty routes to a ribonucleotide world; dispersion and stringency in the decisive selection
Source: RNA. 2018 Aug;24(8):1041–55. doi: 10.1261/rna.066761.118 (PMC6049501; doi:10.1261/rna.066761.118)
Supplement: Supplemental Material [file supp_24_8_1041__index.html]

Eighty routes to a ribonucleotide world; dispersion and stringency in the decisive selection — Supplemental Material 

# Eighty routes to a ribonucleotide world; dispersion and stringency in the decisive selection

## Supplemental Material

- Supplemental\_1\_sporad\_sys\_cross.txt
- Supplemental\_2\_simult\_sys\_cross.txt
- Supplemental\_3\_sporad\_sys\_para.txt
- Supplemental\_4\_simult\_sys\_para.txt
